# A case report of intestinal acute intussusception secondary to a lipoma: Imagery has a great value

**DOI:** 10.1016/j.ijscr.2024.109395

**Published:** 2024-02-16

**Authors:** Souhaib Atri, Ahmed Debiche, Amine Sebai, Azouz Eya, Anis Haddad, Montassar Kacem

**Affiliations:** Hopital la Rabta, Tunis, Tunisia; University of Tunis El Manar Faculty of Medicine of Tunis

**Keywords:** Case report, Acute intestinal intussusception, Lipoma, Adult, Ileocecal intussusception

## Abstract

**Introduction:**

Acute intestinal intussusception (AII) in adults, unlike in children, is a rare manifestation that is frequently secondary to malignant or benign tumors and intestinal intussusception on a lipoma is more exceptional.

**Case presentation:**

We present a rare case of AII caused by a lipoma in a previously healthy 44-year-old man. He experienced severe right lower quadrant pain and constipation. An abdominal CT scan revealed ileocecal intussusception, displaying the classic “sandwich” and “cocarde” images. Additionally, a Caecal lipoma was identified. The patient underwent midline laparotomy, revealing significant small bowel distention upstream of the ileocolic intussusception. Subsequently, a right hemicolectomy with ileocolostomy was performed. Pathological examination confirmed colonic ischemic necrosis attributed to AII originating from a submucosal caecal lipoma.

**Clinical discussion:**

AII is a rare cause of abdominal pain and accounts for 1 to 5 % of adult intestinal obstructions. In adults, an organic cause is found in 70 to 90 % of cases, often secondary to an endoluminal lesion of malignant nature. Pure colonic intussusception on a lipoma is exceptional. CT scan, can show characteristic images and confirms the fatty nature of the lipoma. Surgical intervention is necessary as treatment for intussusception and anatomopathological examination is required for diagnostic confirmation.

**Conclusion:**

Intestinal intussusception caused by an intestinal lipoma is rare. Imaging, mainly ultrasound and CT scan, plays a crucial role in providing a positive and etiological diagnosis of the condition by showing characteristic images. Treatment is always surgical, and there is no place for reduction under radiological control.

## Highlights

• Acute intestinal intussusception in adults is unusual and has diverse etiologies. Furthermore lipomas are rare in the digestive tract, and intestinal intussusception on a lipoma is more exceptional.
• Clinical manifestations appear when lipomas reach a certain size. The symptoms are nonspecific, often presenting as subocclusion, acute obstruction, or nonspecific abdominal syndromes.
• CT scan increases diagnostic sensitivity by demonstrating the pathognomonic features of intussusception. It can diagnose the obstructive syndrome, its mechanism, precise localization, and signs of intestinal compromise, and it can identify the cause.
• Surgical intervention is necessary as anatomopathological examination is required for diagnostic confirmation.
• Reduction under radiological control using hyperpressure is not recommended due to the frequency of underlying organic causes.

## 1 Introduction

Acute intestinal intussusception is a condition primarily seen in infants and young children. Its occurrence in adults is unusual and has diverse etiolog ies. Furthermore lipomas are rare in the digestive tract, and intestinal intussusception on a lipoma is more exceptional. We report a case of ileocecal intussusception on a lipoma. The work has been reported in line with the SCARE criteria [1].

## 2 Case report

A 44-year-old male, previously healthy and non-operated, was admitted to our department with right lower quadrant pain persisting for 12 h, without passage of stool, gas, or vomiting. On examination, the patient was afebrile, with tenderness in the right lower quadrant, and the rest of the abdomen was soft and depressible. A mobile mass was palpated in the right lower quadrant. Laboratory tests showed leukocytosis with a count of 17,000 and negative C-reactive protein (CRP). The patient underwent abdominal CT scan, which revealed distension of the ileal loops with hydro-aerial content, with a maximum measured diameter of 32 mm, and the presence of a fecal sign at the level of the last ileal loop upstream of an ileocecal intussusception measuring 50 × 56 mm with the classic images of the “sandwich” image in longitudinal section, depicting the head of the intussusception, and the “cocarde” image in transverse section without signs of digestive compromise. It revealed also Caecal lipoma measuring 25 × 27 × 40 mm. The patient underwent a midline laparotomy, revealing significant small bowel distention upstream of an ileocolic intussusception with colonic ischemia. A right hemicolectomy with ileocolostomy at the right lower quadrant was performed. Pathological examination showed colonic ischemic necrosis related to AII on a submucosal caecal lipoma. Continuity was restored after 3 months.

## 3 Discussion

Acute intestinal intussusception is a rare cause of abdominal pain and accounts for 1 to 5 % of adult intestinal obstructions [2]. Intestinal intussusception is defined as the telescoping and penetration of an intestinal segment (the intussusceptum) into the distal segment (the intussuscipiens). It is rare in adults and primarily occurs in infants (80 % between 6 months and 2 years). Azar and Berger identified 14 cases of colonic intussusception in adults, including three cases associated with a lipoma, during a retrospective study spanning 30 years at Massachusetts Hospital in Boston [3].While it is often idiopathic in children, in adults, it is frequently secondary to malignant (up to 64 % of cases) or benign tumors. Malignant tumors are the primary etiology. Lipomatous etiology, as in our case, is exceptional [2]. Lipomas are rare lesions in the digestive tract. They predominantly occur in the ileum near the ileocecal valve and proximal jejunum. These tumors, originating beneath the mucosa, develop towards the lumen, pushing the mucosa. Generally, these tumors are asymptomatic [4]. Clinical manifestations appear when lipomas reach a certain size (typically ≥4 cm) [4]. The symptoms are nonspecific, often presenting as subocclusion, acute obstruction, or nonspecific abdominal syndromes (changes in bowel movements, diffuse abdominal pain, gastrointestinal bleeding, etc.), sometimes evolving over several months with or without general deterioration [5]. In our case, the patient was completely asymptomatic until the day of admission when he presented with acute intestinal obstruction and palpable mobile mass in the right iliac fossa, which is unusual in adults. Regardless of the initial clinical presentation, the diagnosis is made in most cases through imaging.

Abdominal X-rays without preparation only allow the diagnosis of small bowel obstruction, rarely revealing the intussusception head as a water-tone mass molded by air from the distal intestinal segment [6].

Ultrasound is effective in diagnosing both intestinal intussusception and a well-defined hyperechoic tumor lesion surrounded by a normal intestinal wall. In longitudinal view, it typically shows a target-shaped image with two hypoechoic outer rings and a central echogenic ring, while in transverse view, it shows a “sandwich” image with three superimposed cylinders representing the intussusception [4,7].

Urgent abdominal CT scan increases diagnostic sensitivity by demonstrating the pathognomonic features of intussusception. It can diagnose the obstructive syndrome, its mechanism (in this case, intussusception), precise localization, and signs of intestinal compromise [4,8], and it can identify the cause (intraluminal or extraluminal mass) [4]. In the case of a lipoma, it reveals an intraluminal lesion with fatty density at the center, surrounded by a digestive wall [8,9]. The two classic images are the “sandwich” image in longitudinal section, depicting the head of the intussusception, and the “cocarde” image in transverse section, showing the intussusception mass.

**Fig. 1:**
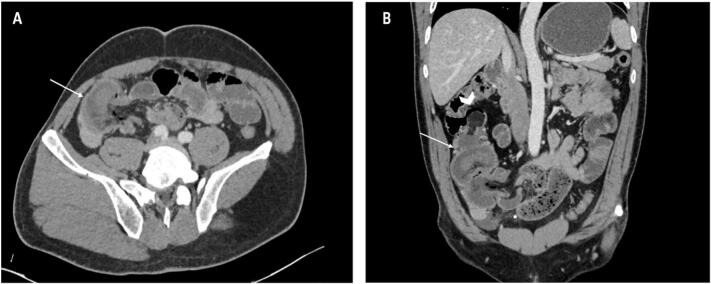
Axial (A) and coronal (B) view of abdominal computed tomography scans demonstrate an ileocolic intussusception (arrow). The leading structure was a fatty dense structure within the bowel lumen that was separate from the mesentery (arrow head). There is a pneumoperitoneum due to small bowel wall necrosis.

In the case of our patient, the CT scan was of great value; it showed the presence of an intussusception sausage-shaped mass with the typical cocoon-like appearance, and it revealed the presence of a lipoma in the cecum as a fatty mass. (See Fig. 1.)

Once the diagnosis is established, surgical intervention is necessary as treatment for intussusception and anatomopathological examination is required for diagnostic confirmation. In some cases, immunohistochemical analysis may be performed (as in lymphoma cases). Reduction under radiological control using hyperpressure is not recommended due to the frequency of underlying organic causes [6,10].

## 4 Conclusion

Intestinal intussusception caused by an intestinal lipoma is rare and directly correlated with the size of the lipoma. Imaging, mainly ultrasound and CT scan, plays a crucial role in providing a positive and etiological diagnosis of the condition by showing characteristic images as the classic images of the “sandwich” image in longitudinal section, depicting the head of the intussusception, and the “cocarde” image in transverse section. It can also confirms the fatty nature of the lipoma. Treatment is always surgical, and there is no place for reduction under radiological control since histological evidence is necessary.

## Consent for publication

Written informed consent was obtained from the patient for publication of this case report and any accompanying images. A copy of the written consent is available for review by the Editor-in-Chief of this journal.

## Ethical approval

Not applicable. Our institution requires no ethical approval for case reports.

## Funding

Not applicable.

All authors read and approved the final manuscript.

The work has been reported in line with the SCARE criteria [1].

## Author contribution

Souhaib Atri: conceptualization, redaction, project manager.

Ahmed Debiche: conceptualization, data curation, redaction, project manager.

Amine Sebai: resources, visualization, supervision.

Azouz Eya: Diagnosed the intussusception by CT scan.

Anis Haddad: Supervision, Validation.

Montassar Kacem: supervision, validation, visualization.

## Guarantor

Debiche Ahmed.

## Research registration number

Not applicable.

## Declaration of competing interest

All authors declare that they have no conflicts of interest.

## Data Availability

Not applicable.
